# Supplementary material for: Intestinal lipid metabolism controls molecular and behavioral immunity through NHR-68 and gut–brain signaling in Caenorhabditis elegans
Source: PLoS Biol. 2026 Sep 29;24(9):e3004026. doi: 10.1371/journal.pbio.3004026 (PMC13649124; doi:10.1371/journal.pbio.3004026)
Supplement: Supplementary file 2 [file pbio.3004026.s010.docx]

Reviewer #1: Overall:
The main claim of this manuscript by Ren *et al* is that *C. elegans* *nhr-68* acts in the intestine to regulate PUFA synthesis, and controls behavioral avoidance and pathogen survival via the AWC neurons. Overall some of the claims are well justified by the data with the 2 important exceptions noted below under major comments. Unfortunately, the level of experimentation, technical and intellectual, analysis is quite basic - the field was reviewing papers at this or level or higher more than 10+ years ago!

Response: We thank the reviewer for the overall assessment and for acknowledging that several aspects of the study are supported by the data.

We respectfully disagree with the characterization that the level of experimentation is basic. We believe our study provides conceptual and technical advances in understanding gut–brain communication during immune responses.

First, we identify an uncharacterized intestine-to-neuron signaling axis in which intestinal lipid metabolic state, regulated by NHR-68, influences an AWC-dependent pathogen-avoidance behavior. Using AWC-ablated animals, we demonstrate that AWC function is required for this behavioral response, thereby linking intestinal metabolism to defined sensory neurons during pathogen exposure.

Second, we show a clear dissociation between behavioral avoidance and survival, where *nhr-68(gk708)* animals display reduced avoidance but enhanced survival upon *P. aeruginosa* infection, indicating that these immune outputs can be independently regulated.

Third, the study combines tissue-specific genetic manipulations and metabolomic profiling to define the role of NHR-68 in regulating intestinal lipid metabolism and host defense behaviors.

In parallel, we show that NHR-68 also affects conserved immune signaling pathways, coordinating behavioral and molecular responses to optimize host protection.

Together, these findings support a model in which intestinal metabolic state communicates with the nervous system to regulate pathogen-avoidance behavior while simultaneously modulating immune responses.

Major comments:

1.      The jump from *nhr-68* to *aex-5* has no clear rationale. Even if LA supplementation bypasses the *nhr-68* phenotype, it could just be parallel or downstream, and have nothing to do with *nhr-68* function itself. Expression level changes of the *fat* genes are not convincing since they do not indicate change in function. Can they show a change in LA levels in *nhr-68* mutants or RNAi? Without this data the link is not plausible.

Response: We thank the reviewer for this thoughtful comment.

The reviewer may have overlooked that we already quantified linoleic acid (LA) levels in *nhr*‑*68(gk708)* animals. These data were presented in Fig 4B and Table S5 of the original submission. To make these results more visually intuitive, we have now reformatted the presentation from a bar graph to a heatmap, which more clearly highlights the reduction of LA in *nhr*‑*68* mutants (updated Fig 4B and Table S5).

We apologize that our rationale for using *aex*‑*5 RNAi* was not sufficiently clear. *aex*‑*5* RNAi served as a reference condition associated with robust pathogen avoidance behavior, rather than a component of the NHR-68 pathway. Prior work has shown that *aex*‑*5* RNAi animals display rapid and strong avoidance behavior (PMID: 31674907), making them a useful system for identifying metabolites associated with effective avoidance. We have revised this explanation in the manuscript. Lines 277-282

Using GC–MS metabolomics, we found that LA is significantly increased in *aex*‑*5* RNAi animals. Consistently, *nhr-68(gk708)* animals show reduced LA levels (Fig 4B, Table S5), whereas *aex-5* RNAi animals show increased LA levels. These reciprocal changes support an association between LA abundance and pathogen-avoidance behavior.

FAT‑3 converts LA into GLA, so loss of *fat*‑*3* activity is expected to cause LA accumulation (Fig 4D). Consistent with this, our GC–MS analysis shows that *fat*‑*3* RNAi animals accumulate LA (new Fig 4G and updated Table S5). This metabolic pattern provides functional evidence that perturbation of the desaturation pathway alters LA abundance. Together, these results outline a model in which intestinal lipid metabolic state, including LA homeostasis regulated by NHR-68 and the desaturation pathway, is associated with pathogen-avoidance behavior. Lines 336-341

2.      What is the mechanism underlying *nhr-68*-in-the-intestine to AWC-controls-the-behavior? Without this, this manuscript is yet another example of 'intestine influences physiology and behavior' for which there are plenty of observations in the literature.

Response: We thank the reviewer for this important comment. We agree that the mechanism linking intestinal NHR-68 activity to neuronal control of avoidance behavior remains an important question.

Our study extends beyond the general observation that intestinal physiology influences behavior. Previous studies have provided important insights into gut–brain communication and demonstrated that intestinal state can affect neural and behavioral outputs. Our work differs in that it identifies a specific intestinal metabolic pathway involving NHR-68, the fatty-acid desaturases FAT-2 and FAT-3, and LA that regulates pathogen-avoidance behavior. Thus, rather than establishing a general link between intestinal physiology and behavior, our study defines a molecularly defined lipid metabolic pathway linking intestinal fatty-acid homeostasis to behavioral immune regulation.

Our data support a model in which NHR-68 functions in the intestine to regulate polyunsaturated fatty-acid homeostasis through the fatty-acid desaturation pathway. Genetic perturbations that alter LA levels, including *nhr-68* deletion, *fat-2* RNAi, *fat-3* RNAi, and LA supplementation, produce corresponding changes in pathogen-avoidance behavior. Furthermore, AWC neurons are required for both NHR-68-dependent and LA-associated avoidance responses, linking intestinal lipid metabolism to a defined sensory circuit.

Importantly, our current data do not demonstrate that LA directly acts on AWC neurons. Rather, we favor a model in which alterations in intestinal fatty-acid metabolic state generate a gut-derived signal that modulates neuronal circuits controlling avoidance behavior, with AWC representing a key component of this circuit.

We have revised the manuscript to clarify this interpretation and to avoid implying direct action of LA on AWC neurons. Specifically, we have updated the conclusion sentences in Results and the Discussion to reflect this revised interpretation. Lines 355-357, 372-374 and 482-505

Thus, our findings define a mechanistically supported intestine-to-neuron pathway in which intestinal NHR-68-dependent lipid metabolism regulates an AWC-dependent behavioral response, while the molecular nature of the gut-to-brain signal remains to be determined.

Minor Comments:
1.      Line 48 in introduction. Please cite the many papers from Srinivasan, Wang, Sengupta and other labs that have already demonstrated interorgan communication and the role of the intestine therein. 

Response: We have revised accordingly to include additional citations from the Srinivasan, Wang, Sengupta, and other laboratories. Lines 52-53

Reviewer #2: In this study, Ren *et al.* describe a role for two nuclear hormone receptors, nhr-68 and nhr-110, in the response of *C. elegans* to infection by *Pseudomonas aeruginosa* (PA14). These genes were identified based on their induction of an immune marker gene, *clec-60*, typically upregulated upon PA14 exposure. The authors report an intriguing dichotomy: mutants for both nhrs exhibit defective/reduced pathogen avoidance yet display enhanced survival following infection. This finding raises important questions regarding the relationship between neural circuits that mediate pathogen avoidance and molecular mechanisms governing immune defense. The authors further show that *nhr-68* acts in the intestine to impair pathogen avoidance by repressing levels of a polyunsaturated fatty acid linoleic acid (LA), suggesting intestine-to-neuron crosstalk through metabolic signaling. Overall, this study is founded on compelling, exciting observations and with careful investigation has the potential to provide valuable insights into metabolic regulation of host defense and, importantly, into the links between 'behavioral' 'and 'molecular' facets of immunity. The main concern is that this conceptual connection is not sufficiently explored, and deeper investigation is required to fully realize the novelty and significance suggested by the data.

Response: We thank the reviewer for this insightful comment. In response, we have performed additional survival analyses in neuronal ablation backgrounds and in lipid metabolic perturbation conditions (including LA supplementation and *fat-3* RNAi), and expanded the Discussion to further address the behavioral–immune relationship.

These analyses demonstrate that pathogen-avoidance behavior and host survival are mechanistically separable outputs of NHR-68 function: avoidance depends on an AWC- and LA-associated pathway, whereas survival is independent of both AWC neurons and LA levels.

Together, these results strengthen the manuscript by refining the mechanistic framework of NHR-68–mediated host defense and clarifying the dissociation between behavioral and physiological immunity.

Major points

1. One of the most striking observations in this study is that *nhr-68* and *nhr-10* mutants show markedly impaired pathogen avoidance yet survive significantly longer upon infection than wild-type animals. Thus, reduced avoidance does not compromise immune defense and is, paradoxically, associated with enhanced survival. It remains unclear whether these phenotypes are mechanistically linked or represent independent functions. Is the enhanced immune response compensatory, or does it arise from a shared regulatory pathway? The rescue of both phenotypes by the *nhr-68* reporter construct (Fig 2C) suggests a connection, but this relationship requires more rigorous investigation and deeper discussion. For example, in the neuronal rescue experiments shown in Fig 5, it is essential to document the corresponding PA14 survival dynamics. Similarly, for the LA supplementation and desaturase mutant experiments (Fig 4), survival on PA14 should be assessed in the specific context of these *nhr*s. Although PA14-infection survival effects of lipid-metabolism mutants and fatty-acid supplementation have been reported previously, understanding how these manipulations influence behavior and survival within the *nhr-68/nhr-10* framework is critical. A more explicit discussion of this apparent behavioral- immune dichotomy would also be valuable.

Response: Previous studies have shown that impaired pathogen avoidance can coexist with enhanced survival, for example in *nol-6*–deficient animals, which exhibit reduced avoidance yet increased resistance to *P. aeruginosa* due to decreased intestinal colonization and activation of internal immune defenses (PMID: 19763173; PMID: 30827896). In these contexts, enhanced survival arises from effective internal immune control rather than intact avoidance behavior.

To directly address this in our study, we performed PA14 survival assays in both the neuron-ablation experiments (new Figs S10A-S10D) and the LA supplementation/desaturase mutant experiments (new Figs S8A). Under each condition tested, manipulations that alter avoidance behavior do not significantly change the enhanced survival phenotype of *nhr-68(gk708)* animals. Specifically, ablation of AWC, AWB, ASE, or ASI neurons did not alter the enhanced survival phenotype of *nhr-68(gk708)* animals (Fig S10A–S10D). In parallel, neither exogenous linoleic acid (LA) supplementation nor *fat-3* RNAi–induced LA accumulation altered the increased survival of *nhr-68(gk708)* animals (Fig S8A–S8B). Together, these results demonstrate a dissociation between pathogen avoidance behavior and host survival in *nhr-68(gk708)* animals. This finding supports the existence of parallel regulatory mechanisms governing behavioral and physiological responses during infection. Lines 322-325, 342-346 and 400-411

2. The relationship between *nhr-68* and *nhr-10* is intriguing but insufficiently explored. While *clec-60* expression and survival data suggest parallel functions, the pathogen-avoidance phenotypes are more consistent with a shared pathway. It is therefore surprising that tissue-specific rescue experiments were performed for both mutants, whereas transcriptomic analyses- which may be even more informative in this context- were not extended comparably. This issue warrants further investigation. Do the two mutants share similarities or differences in their RNA-seq profiles on OP50 and PA14? Do these genes regulate each other's expression? Can the *nhr-68* construct rescue avoidance or survival phenotypes in *nhr-10* mutants? Addressing these questions would substantially clarify the functional relationship between the two receptors and help resolve the issue raised above.

Response: We appreciate the reviewer's comments regarding the relationship between NHR-10 and NHR-68. Previous studies demonstrated that NHR-10 transcriptionally activates *nhr-68* and that the two factors function in a regulatory module controlling propionate-responsive genes (PMID: 30625328). In this model, *nhr-68*::GFP expression is abolished upon *nhr-10* RNAi, indicating that *nhr-68* expression depends on NHR-10 activity.

To characterize their functional relationship, we quantified NHR-68–regulated immune gene *clec-60* in wild-type, *nhr-10(tm4695)*, *nhr-68(gk708)*, and double mutant animals (Fig 1B). These analyses show that both NHR-10 and NHR-68 contribute to regulation of *clec-60* expression and act additively in controlling immune gene expression.

In addition, qRT–PCR analysis of multiple NHR-68–regulated immune genes shown in Fig. 3C demonstrated that most genes were also upregulated in *nhr-10(tm4695)* animals (new Fig S6B), consistent with shared regulation of immune gene targets by both NHR-10 and NHR-68. Line 269-273

Because the previously established feed-forward loop requires both NHR-10 and NHR-68 for downstream transcriptional activation, expression of *nhr-68* alone would not be expected to rescue the loss of *nhr-10* function. Therefore, rescue of *nhr-10* mutants with the *nhr-68* transgene was not pursued.

We have added a discussion of the NHR-10–NHR-68 feed-forward loop and its relevance to immune regulation. Lines 528-539

3. Is *nhr-68* expressed in AWC or other sensory neurons implicated in pathogen avoidance? How does neuronal versus intestinal expression of *nhr-68* influence survival on PA14? Additionally, is there a role for hypodermal expression in these phenotypes?

Response: As shown in Fig 2, *nhr-68* is predominantly expressed in the intestine. In addition, we observe faint expression in the hypodermis, a single head neuron, and several head muscle cells. We do not detect expression in AWC neurons.

To determine the tissue in which *nhr-68* functions to regulate avoidance behavior and host survival during *P. aeruginosa* infection, we performed tissue-specific RNAi experiments. Intestine-specific knockdown of *nhr-68* significantly reduced avoidance behavior and increased survival on *P. aeruginosa* (Fig 6B and new Fig 6F), whereas neuron-specific knockdown did not produce a detectable effect (Fig 6C and new Fig 6G). These results indicate that intestinal *nhr-68* is required for host defense during *P. aeruginosa* infection.

Consistent with these findings, intestinal expression of *nhr-68* was sufficient to rescue the reduced avoidance behavior of *nhr-68(gk708)* animals (Fig 6D). Moreover, intestinal expression of *nhr-68* partially rescued the pathogen susceptibility of *nhr-68(gk708)* animals (Fig 6H), further supporting a major role of intestinal *nhr-68* in host defense against *P. aeruginosa*. Although a contribution from the low-level hypodermal expression cannot be excluded, our data demonstrate that intestinal *nhr-68* is sufficient for these phenotypes, whereas neuronal expression is not required. Lines 448-453

4. The rationale for performing GC-MS on *aex-5* RNAi animals to implicate LA is hazy and insufficiently justified. Why was fatty-acid profiling not performed directly on *nhr-68* mutants? Given the functional roles uncovered for LA and the desaturase enzymes, it is particularly important to establish the fatty-acid composition of these mutants themselves.

Response: We thank the reviewer for this insightful comment. *aex*‑*5* RNAi served as a reference condition associated with robust pathogen avoidance behavior, rather than a component of the NHR-68 pathway. Prior work has shown that *aex*‑*5* RNAi animals display rapid and strong avoidance behavior, making them a useful system for identifying metabolites associated with effective avoidance. We have revised this explanation in the manuscript. Lines 277-282

We further clarify the rationale and the order of these analyses in the revised manuscript. The GC–MS metabolomic profiling of *aex-5* RNAi animals was generated previously, in the course of our work on this strong-avoidance state. Reasoning that a metabolite that promotes avoidance should be enriched in a strongly avoiding background, we used this existing dataset as an unbiased screen, which pointed to linoleic acid (LA). Because *nhr-68(gk708)* animals avoid poorly, we then profiled LA directly in these mutants.

We performed targeted LC–HRMS quantification of LA together with its upstream and downstream fatty acid species in both WT and *nhr-68(gk708)* animals. LA levels in *nhr-68(gk708) animals* were significantly reduced (updated Fig 4B and Table S5), providing direct evidence of altered LA homeostasis in *nhr-68(gk708)* animals. Lines 310-316

Given that LA was the metabolite of primary interest identified in our study and had already been quantitatively assessed by targeted LC–HRMS, additional untargeted GC–MS profiling of *nhr-68* mutants was not performed. Instead, we additionally performed metabolic profiling of *fat-3* RNAi animals. Based on the PUFA biosynthetic pathway (Fig 4D), *fat-3* knockdown is predicted to result in LA accumulation, which showed a significant increase in LA abundance (new Fig 4G and updated Table S5). Together, these complementary genetic and metabolic data support a model in which NHR-68 regulates LA homeostasis through the FAT-3–dependent desaturation pathway. Lines 336-341

5. The comparison between *nhr-68*-dependent transcriptomes and previously published PA14 datasets requires greater detail. For example, do mutants grown on OP50 exhibit significant overlap with genes that are induced or repressed in wild-type animals upon PA14 infection? do shared features include both upregulated and downregulated gene categories? What about overlaps with other metabolic regulatory TFs, nhrs and others, that have been implicated in PA14 response?

Response: We thank the reviewer for this suggestion. We performed comparative analyses of NHR-68–dependent gene sets with PA14-regulated transcriptomes (updated Tables S2 and updated Table S3), revealing overlaps with both infection-induced and infection-repressed genes, as well as immune and metabolic regulatory transcriptional programs. These results have been incorporated into the revised manuscript. Lines 259-265

Minor points

6. Do wild-type animals exposed to PA14 exhibit changes in linoleic acid levels, or in nhr-68/nhr-110 mRNA or protein abundance?

Response: We measured linoleic acid levels in wild-type animals exposed to *P. aeruginosa* PA14 by GC/MS and observed a modest increase relative to animals maintained on *E. coli* (new Fig 4G; updated Table S5). Lines 347-352

In addition, qRT–PCR analysis showed that both *nhr-68* and *nhr-10* transcript levels were modestly increased upon PA14 exposure in wild-type animals (Fig S6A). Lines 265-258

These results indicate that pathogen exposure is associated with coordinated changes in both intestinal lipid metabolism and the expression of NHR transcriptional regulators.

7. Additional background information is needed on lipid-metabolic regulation of immune responses, particularly regarding desaturation and elongation pathway mutants and their PA14 phenotypes, as well as prior supplementation studies. Other immune regulators beyond daf-16 and pmk-1 should also be discussed. Expanding this material would substantially strengthen the Introduction and Discussion, which are currently rather brief.

Response: We thank the reviewer for this suggestion. We have expanded the Introduction to include additional background on lipid-metabolic regulation of immunity, including desaturation and elongation pathway mutants and fatty-acid supplementation studies. Lines 69-72

We also expanded the Discussion to include other immune regulators beyond PMK-1 and DAF-16 in a broader context. Lines 511-518

9. Figure legends should include mean survival values for the survival curves shown in panels, along with clearer descriptions of statistical analyses, including which particular comparisons the P values refer to.
We have updated all survival curve figure legends to include mean survival values and clarified the statistical analyses, specifying which comparisons the P values refer to. Updated figure legends for Figs 1C, 2C, 3D-3F, 6E-6H, S3B and S8A-8B

10. In Fig 3C, nhr-68 is mislabeled as nhr-86.
We corrected the label in Fig 3C: *nhr-86* has been changed to *nhr-68*.

11. Are the nhr alleles used in this study null mutations? A brief description of the molecular lesions and any previously reported phenotypes would help orient the reader.
We have briefly described *nhr-10(tm4695)* *and nhr-68(gk708)* in the Results section as deletion alleles predicted to cause loss of function. Lines 138-139

In addition, we have included the precise deletion sizes in Table S6 Strains.

Reviewer #3:

Ren, Aballay and colleagues studied how immune response and avoidance behavior under pathogen infection are coordinated, focusing on the nuclear hormone receptor (NHR) family of transcription factors in the nematode *C. elegans*. They found that mutations of *nhr-68* reduced behavioral avoidance but enhanced immune response to *Pseudomonas aeruginosa*, a natural *C. elegans* pathogen. *nhr-68* acted in parallel to *nhr-10*, and it functioned in the intestine. With RNA-seq experiments, they found that *nhr-68* repressed immune genes related to the PMK-1/MAPK and DAF-16/FOXO pathways. Moreover, they performed lipidomics analysis and showed that *nhr-68* promoted the production of linoleic acid (LA), which could potentially stimulate behavioral avoidance for *Pseudomonas*. The authors then went on to show that the AWC chemosensory neurons mediated avoidance to Pseudomonas downstream of *nhr-68* and LA. Taking all these findings together, they proposed a gut-brain signaling model for NHR-68-mediated pathogen avoidance behavior.
        
The most important conceptual advance of this study lies in the discovery of a transcription factor that acts in an immunity tissue (the intestine) to coordinate two aspects of immunity (molecular and behavioral). The identification of LA as a peripheral signal for behavioral modulation expands the repertoire of peripheral signals that remodel the functions of the nervous system. There are several important mechanistic questions that were not addressed in the study, making the study a bit short of significant conceptual advance. To improve the manuscript further, the authors should address a few questions listed below.

Response: We thank Reviewer #3 for the thoughtful and constructive comments and for recognizing the conceptual advance of our study. In response, we performed several additional experiments and substantially revised the manuscript to clarify key mechanistic questions and expand the Discussion. Together, these revisions strengthen both the mechanistic framework and the overall clarity of the manuscript.

Major:

1. A major unanswered question for the study is whether LA production is indeed increased under *Pseudomonas* infection, which is a vaguely hinted but never explicitly expressed idea throughout the study that lacks experimental support. Therefore, the authors should perform metabolome analysis to address this critical issue, and test whether this *Pseudomonas*-induced LA increase (if demonstrated experimentally) is *nhr-68*- and *fat-3*-dependent.

Response: We thank the reviewer for this important suggestion. To address this question, we performed targeted GC/MS analysis of linoleic acid (LA) abundance in animals exposed to either *E. coli* or *P. aeruginosa* PA14. In the absence of infection, *fat-3* RNAi animals showed increased LA levels significantly compared with controls (new Fig 4G and updated Table S5). We further found that PA14 exposure increased LA levels in wild-type animals (Fig 4G and Table S5). In contrast, no further increase in LA levels was observed in *fat-3* RNAi animals under infection conditions, which already displayed elevated basal LA levels (Fig 4G and Table S5), supporting a role for FAT-3 in regulating LA homeostasis during infection. These new data have been added to the revised manuscript. Lines 336-346

In addition, as described in our response above (Reviewer #2, Major Comment 4), we previously performed targeted LC–HRMS quantification of LA and found that LA levels were significantly reduced in *nhr-68(gk708)* animals (updated Fig 4B and Table S5), providing direct evidence that NHR-68 regulates LA homeostasis. Lines 310-313

Together, these results suggest that pathogen-induced changes in LA abundance and basal LA homeostasis are controlled through a regulatory framework involving both NHR-68 and FAT-3.

2. A related question is how NHR-68 inhibits *fat-3* expression. Is *fat-3* a direct target for repression by NHR-68? Any known sequence motif in the promoter of *fat-3* for NHR-68 binding?

Response: We thank the reviewer for this insightful question. At present, there is no experimentally defined DNA-binding motif for NHR-68 in *C. elegans*. Only a limited number of nematode nuclear hormone receptors have been characterized at the level of genomic binding sites, and NHRs often recognize diverse or context-dependent DNA sequences, making it difficult to predict NHR-68 binding motifs based on related family members. Our qRT-PCR analysis showed that *fat-3* expression is elevated in *nhr-68(gk708*) animals, indicating that NHR-68 negatively regulates *fat-3* expression. However, the current data does not distinguish whether this regulation occurs through direct promoter binding or indirectly through intermediate regulatory factors. We have added a brief discussion of this limitation in the revised manuscript (Lines 478-481).

3. Another key unresolved issue is how the AWC neuron is regulated by LA. This includes (1) identification of the receptor for LA; (2) demonstration that the LA receptor is expressed and acts in AWC; and (3) the physiological or molecular properties of AWC that are altered by LA and/or the LA receptor. Without these discoveries, this gut-brain signaling circuit lacks essential mechanistic detail. A less likely mechanism is that LA is directly incorporated into the membrane of AWC, leading to a change in neuronal membrane properties. However, this possibility fails to explain why AWC is selectively targeted by LA.

Response: We thank the reviewer for this thoughtful comment. We agree that the molecular mechanism by which intestinal lipid metabolism influences neuronal activity remains unresolved.

Our current data demonstrate that intestinal NHR-68 regulates polyunsaturated fatty-acid metabolism and that AWC neurons are required for the resulting pathogen-avoidance behavior. However, we do not have evidence that linoleic acid (LA) directly targets AWC neurons, nor do we propose that AWC is necessarily the primary site of LA action. Therefore, identification of an LA receptor in AWC is beyond the scope of the conclusions supported by the current study.

We have revised the manuscript to clarify that our findings support an intestine-to-neuron signaling pathway in which alterations in intestinal fatty-acid metabolism influence an AWC-dependent behavioral circuit through an indirect signaling mechanism. Specifically, we now emphasize that NHR-68-dependent regulation of intestinal lipid metabolism acts upstream of an AWC-dependent behavioral circuit, without assigning the molecular identity of the gut-derived signal. This signal may involve lipid-derived metabolites, neuroendocrine factors, or other intercellular signaling mechanisms, which will require future studies to resolve.

Specifically, we have updated the conclusion sentences in the Results and the Discussion to reflect this revised interpretation. Lines 355-357, 372-374 and 482-505

Thus, our findings define a mechanistically supported intestine-to-neuron pathway in which intestinal NHR-68-dependent lipid metabolism regulates an AWC-dependent behavioral response, while the molecular nature of the gut-to-brain signal remains to be determined.

4. What are some of the neurons that express *nhr-68* in the head? Is *nhr-68* expressed in AWC?

Response: We observed weak expression in a single head neuron, but were unable to confidently identify its neuronal identity based on position and morphology alone. Therefore, we cannot determine whether this neuron corresponds to a specific neuronal subtype. Although we cannot exclude low-level expression in individual neurons, our functional analyses indicate that neuronal *nhr-68* is not required for pathogen avoidance behavior (Fig 6).

5. Does upregulation of immune genes in the *nhr-68* mutant compromise behavioral avoidance? In other words, the authors should explore whether enhanced immunity and decreased avoidance in the *nhr-68* mutant are causal of each other, or they represent two independent outcomes of the *nhr-68* mutation. The idea of compensation or trade-off could explain why increased immunity impairs behavioral responses. Alternatively, these two outcomes are independent of each other; but in this scenario, it is more difficult to explain why these two opposite outcomes are coordinated by a single gene.

Response: Our new data support the conclusion that enhanced immunity and reduced avoidance in *nhr-68(gk708)* animals represent parallel but mechanistically separable outcomes, rather than a direct trade-off in which increased immune activation suppresses behavioral responses.

Previous studies have demonstrated that impaired pathogen avoidance can coexist with enhanced survival. For example, *nol-6*–deficient animals exhibit reduced avoidance of *P. aeruginosa* yet increased resistance to infection due to reduced intestinal colonization and activation of internal defense mechanisms (PMID: 19763173; PMID: 30827896). These studies indicate that behavioral avoidance and immune protection can be coordinated but function through distinct downstream pathways.

To directly address this issue in our system, we performed additional PA14 survival assays in the contexts that restore or alter avoidance behavior. We assessed survival in neuron-ablated animals as well as under metabolic perturbations that alter linoleic acid levels, including LA supplementation and *fat-3* RNAi. Although these manipulations affect avoidance-related phenotypes, none altered the enhanced survival of *nhr-68(gk708)* animals (new Figs S8A-S8B and S10A-S10D). Under each condition tested, modulation of the avoidance pathway does not significantly affect the survival phenotype of *nhr-68(gk708)* animals. Lines 322-325, 342-346 and 400-411

Together, our results support a model in which NHR-68 coordinates pathogen-avoidance behavior and survival through separable downstream outputs, with avoidance requiring an intestinal lipid-dependent AWC circuit, while survival is regulated independently of this pathway.

6. Although the discovery of LA as one peripheral signal for pathogen avoidance is exciting, it is not immediately clear why *aex-5* RNAi was used for this discovery. I understand that defective defecation motor program (DMP) induces avoidance behavior. However, the connection in the logics of the DMP model and the *Pseudomonas avoidance* model needs to be better described. In the methods, it was not mentioned whether the *aex-5* RNAi animals were grown on OP50 or *Pseudomonas*; this should be made clear.

Response: We would like to clarify that our intention was not to suggest a direct mechanistic link between *aex-5* and *nhr-68*, nor to implicate the defecation motor program (DMP) pathway in NHR-68 function. Rather, *aex-5* RNAi was used as a previously established model that exhibits robust pathogen avoidance behavior upon disruption of the DMP pathway (PMID: 31674907), and therefore provides a useful context for identifying metabolites associated with effective avoidance. We have revised this explanation in the manuscript. Lines 277-282

Consistent with this rationale, GC–MS metabolomic profiling identified linoleic acid as a metabolite enriched in *aex-5* RNAi animals. As described in our response to Review #1 Major Comment 1 and Review #2 Major Comment 4, targeted LC–HRMS analysis further demonstrated that LA levels are significantly reduced in *nhr-68(gk708)* animals (updated Fig 4B and updated Table S5), whereas *fat-3* RNAi and exogenous LA supplementation increase LA levels (new Fig 4G and updated Table S5) and modulate pathogen-avoidance behavior (Fig 4H). Together, these complementary genetic and metabolic analyses support an association between LA abundance and pathogen-avoidance behavior, and establish its functional link to NHR-68 through direct measurement in *nhr-68* mutants. Lines 310-313 and 342-352

We have clarified the experimental conditions used for metabolomic analysis in the Methods section. Specifically, *aex-5* RNAi animals were grown on *E. coli* HT115 and collected prior to *P. aeruginosa* exposure for GC–MS analysis. Lines 733-735

7. I am a bit surprised that LA also enhances *Pseudomonas* avoidance in the wild type (Figure 4C). I would have expected that in the wild type, which has a functional *nhr-68* gene, the production of LA is high enough to drive optimal avoidance. The authors should test whether LA itself is repulsive for *C. elegans*, a function that is unrelated to learning (*Pseudomonas* avoidance in adult *C. elegans* is learned, not innate).

Response: To address the concern that linoleic acid (LA) might function as a direct repellent and thereby artificially enhance *P.* avoidance in wild-type animals, we performed bacterial two-choice preference assays using LA-supplemented and vehicle-treated bacterial lawns. In these assays, worms did not show preferential avoidance of LA-supplemented *E. coli* or *P. aeruginosa* lawns compared with vehicle controls. These results indicate that LA does not act as an innate aversive cue in *C. elegans*, but instead modulates pathogen avoidance in a context-dependent manner. We have added a statement in the revised manuscript noting that a direct aversive effect of LA was ruled out by choice assays (Fig S7B). Lines 316-317

8. In Figure 4A, there are other metabolites that are increased in the *aex-5* RNAi animals, such as GABA and 3-phosphoglycerate. What are their levels in the *nhr-68* mutant? Do they show similar changes to those of LA?

Response: Since we already performed LC–HRMS analysis in *nhr-68(gk708)* animals, we did not conduct additional GC–MS profiling in *nhr-68* animals. Instead, we performed GC–MS analysis in *fat-3* RNAi animals, which accumulate LA (Fig 4D and updated Fig 4G).

In this dataset, we detected changes in additional metabolites, including GABA and 3-phosphoglycerate (Table S5). However, these metabolites were not included in the main text, as they were not further pursued in the current study. In *fat-3* RNAi animals, GABA showed a modest increase, whereas 3-phosphoglycerate did not exhibit significant changes (updated Table S5).

These observations suggest that *fat-3* perturbation induces selective metabolic alterations, with partial overlap to metabolites affected in the *aex-5* RNAi condition, while LA remains the most robust and consistent change across conditions.

9. *nhr-10* and *nhr-68* single and double mutants show decreased avoidance to *Pseudomonas aeruginosa* in the lawn occupancy assay. It is important to provide experimental evidence that this is not a consequence of reduced locomotion ability of these mutants.

Response: Locomotion assays confirmed that the reduced avoidance phenotype observed in *nhr-10(tm4695)*, *nhr-68(gk708)*, and double mutant animals was not due to impaired locomotion ability (new Fig S2B). We have added this information to the revised manuscript. Lines 146-148

Minor:

1. What are the known roles of other nhr genes from the RNAi screen (for example, *nhr-77*, *nhr-130*, *nhr-222*, etc)? The authors may want to discuss a little bit on this.
Response: We have added a brief discussion of the other NHRs identified in our RNAi screen in the revised manuscript, noting that most remain poorly characterized but belong to a family broadly implicated in metabolic and stress-responsive regulation. Lines 540-551

2. In Figure 1A legend, "…(n=8); data are representative of three independent experiments." This is confusing; please clarify what the sample size is for data presented in Figure 1A.
Response: Figure 1A represents data from three independent experiments, each including 8 animals. We have clarified this in the figure legend in the revised manuscript. Lines 121-122

3. Figure 3D should be incorporated into Figure 3E and Figure 3F, so that the readers understand how *pmk-1* and *daf-16* contribute to the extended survival of the nhr-68 mutants under *Pseudomonas* infection.
Response: We thank the reviewer for this helpful suggestion. We agree that integrating these data could provide an alternative visualization of the results. However, we chose to present Fig 3D–3F as separate panels to clearly distinguish the effects of *pmk-1* and *daf-16* perturbations on *nhr-68*-dependent survival, which allows for more direct comparison of each condition. We have revised the figure legends to clarify this relationship and to guide the reader in interpreting these results. Lines 225-228

4. Line 315-316: Consistent with AWC, AWB and ASI being required for *Pseudomonas* avoidance, ablation of each of these three neurons reduced avoidance (Figures 5A-C, the empty vector groups of cell ablation). The authors could describe this result here to strengthen the importance of these neurons in *Pseudomonas* avoidance and also highlight the fact that only AWC is specific for the *nhr-68*/LA pathway.

Response: We have revised the text to note that AWC, AWB, and ASI each contribute to *Pseudomonas* avoidance, while only AWC is specifically linked to the *nhr-68*/LA pathway. Lines 361-363

5. Could the authors provide positive control data showing that MAH677 is sensitized for RNAi of neuronal genes? This is important for excluding the possibility that gene knockdown in this strain is inefficient.

Response: We thank the reviewer for this suggestion. To better justify the use of MAH677, we have now added a brief description and references indicating that MAH677 is an established neuronal RNAi-sensitized strain that supports efficient knockdown of neuronal genes and robust behavioral phenotypes. Therefore, we did not perform additional positive control experiments. Lines 418-420

6. There are several recent papers that explore peripheral to neuron signaling regarding behaviors or lipid metabolism (PMID 38092521, PMID 38520690, PMID 40252647, PMID 40425807). The authors may consider discussing some of these in their study.

Response: We have revised the Discussion to incorporate recent studies on peripheral-to-neuron signaling and lipid-mediated regulation of behavior (PMIDs: 38092521, 38520690, 40252647, 40425807). Lines 490-498

7. Please include "behavior" in the title so that it more faithfully reflects the contents of the study.
Response: We thank the reviewer for this suggestion. After consideration, we have retained the current title. The overall immune response comprises both molecular immunity (activation of microbial-killing pathways) and behavioral immunity (avoidance of pathogens). Because "immune response" already encompasses both arms of defense, we have kept the title general rather than singling out the behavioral component. The Abstract and Introduction make explicit that the study addresses both the molecular and behavioral aspects of the overall response.

Reviewer #4: In the manuscript "Intestinal lipid metabolism controls immune response through NHR-68 and gut-brain signaling in *C. elegans*", Ren and colleagues identify a lipid metabolism pathway that impacts behavioral avoidance of pathogens. The strength of this manuscript lies in the identification of a nuclear hormone receptor, acting in the gut, which transcriptionally regulates genes that alter levels of polyunsaturated fatty acids, which, in turn, affect avoidance behavior. Particularly strong are the experiments demonstrating that increasing levels of a specific PUFA, linoleic acid (LA), via direct supplementation or by knockdown of the desaturase *fat-3*, rescues the behavioral phenotypes of mutants (like *nhr-68*) that have lower levels of LA. They also identify neurons involved in the pathogen behavioral avoidance in this context and provide evidence suggesting that LA signals to the AWC sensory neuron to drive this avoidance behavior. The identification of a lipid metabolism and intertissue signaling pathway involved in gut-brain communication is novel and of broad interest. However, as it stands, the manuscript lacks some key evidence to support their claim that LA signals from the gut to the AWC neuron to drive these behavioral effects.

We thank Reviewer #4 for the thoughtful and constructive comments. We appreciate the reviewer’s suggestions regarding the mechanistic relationship between intestinal lipid metabolism, neuronal regulation of pathogen avoidance, and the distinction between behavioral and molecular immunity. In response, we performed several additional experiments and substantially revised the manuscript. Specifically, we clarified the role of linoleic acid in NHR-68–dependent behavioral regulation, expanded the analysis of fatty-acid metabolism through genetic and metabolic approaches, added survival assays to distinguish behavioral and physiological outcomes, refined the interpretation of the intestine-to-neuron pathway to avoid overstatement regarding direct LA signaling to AWC neurons, and expanded the Discussion to incorporate recent studies on peripheral-to-neuron communication. Together, these revisions strengthen the mechanistic framework, clarify the scope of our conclusions, and further support a model in which intestinal NHR-68 coordinates behavioral and molecular host defenses through distinct downstream pathways.

Major concerns:

1)      The specific role of AWC in avoidance in this context. It's clear from earlier work from the authors' lab as well as other labs (Zhang, Kim etc…), that multiple sensory neurons are involved in PA14 avoidance behavior. How do the authors square the results here showing that AWC is the only neuron showing a strong defect in avoidance? Also, because AWC(-) animals appear to have the strongest phenotype of the ablation strains, it seems plausible that there is a ceiling effect here and that AWC is simply essential for avoidance. The effect of LA should be tested in the other neuron ablation strains, and if possible, they should identify whether there are observable changes in AWC activity. Because *nhr-68* appears to act specifically in the gut, it's unclear how this would result in changes specific to AWC.

Relatedly, in line 321, "Among the neuron-ablated strains, only AWC(-) animals showed a significant reduction in avoidance behavior upon nhr-68 knockdown, similar to the phenotype of *nhr-68* RNAi animals in a wild-type background (Figs 5A and S7A). Ablation of AWB, ASI, or ASE neurons had no effect on the avoidance phenotype of *nhr 68*-deficient animals (Figs 5B-5D, S7B-S7D)." This can't be what the authors meant to state here. To me, the data essentially show the opposite, that only AWC(-) animals are unaffected by nhr-68 knockdown?

Response: For the LA supplementation experiments, we have extended the analysis to all neuron ablation strains and revised the text accordingly (Fig 5E, S9E, new Figs 5F–5H and new Figs S9F–S9H). We show that LA enhances avoidance behavior in WT, AWB(-), ASI(-), and ASE(-) animals, but fails to rescue the avoidance defect in AWC(-) animals. Together, these results indicate that AWC is specifically required for both NHR-68-dependent and LA-mediated behavioral responses to *P. aeruginosa*. The corresponding text has been updated in Lines 392-396

For the neuron ablation analysis, we have corrected the interpretation of the *nhr-68* RNAi phenotype across different neuronal ablation backgrounds. We now state that only AWC(-) animals fail to exhibit an additional reduction in avoidance behavior upon *nhr-68* knockdown, whereas AWB(-), ASI(-), and ASE(-) animals retain significant reductions, indicating that NHR-68-dependent modulation specifically requires AWC. Lines 368-370

2)      The specific role of LA as a gut-to-brain signal. While the LA supplementation experiments are strong, and clearly shows that LA acts downstream of nhr-68, it's unclear where, when and how LA acts. Does it signal directly in the gut or is it further desaturated or oxidized to produce something else that is active? Given the many hours between supplementation and the behavioral outcome, the LA signal could be many steps upstream of a change in the sensory neurons. I would suggest determining whether additional *fat* metabolism genes alter the observed phenotypes, such as whether e.g. *fat-1* (downstream) suppresses the *fat-3* RNAi or LA supplementation or whether *fat-2* (upstream) suppresses the *fat-3* phenotypes. Given that the authors seem to imply that the LA accumulation in *aex-5* mutants is related to the phenotype of *nhr-68* mutants, does *fat-2* RNAi suppress the phenotype of *aex-5*?

Response: For the mechanism underlying LA-mediated gut-to-brain communication, we have revised the manuscript to better clarify the working model. Rather than implying direct signaling from LA to AWC neurons, we favor a model in which alterations in intestinal fatty-acid metabolism generate a gut-derived metabolic signal that subsequently modulates neuronal circuits controlling avoidance behavior, with AWC representing a key downstream component of this circuit. Whether this communication occurs through LA itself, another lipid-derived metabolite, neuroendocrine signaling, or additional intermediary cells remains an important area for future investigation. The corresponding text has been updated in the Results and Discussion. Lines 355-357, 372-374 and 482-505

Regarding additional fatty-acid metabolism genes, we further clarified that the existing data support regulation of LA homeostasis through the desaturase pathway downstream of NHR-68. Specifically, *fat-3* RNAi increased LA abundance (new Fig 4G and updated Table S5) and enhanced pathogen avoidance (Figs 4H and S7D), whereas reducing LA synthesis by *fat-2* RNAi significantly decreased pathogen avoidance, phenocopying the defect observed in *nhr-68(gk708)* animals (Figs 4I and S7E). The corresponding text has been updated. Lines 342-346 and 353-355

Finally, although *aex-5* RNAi was useful as a metabolic model to identify candidate lipid signals, *aex-5*-mediated avoidance is mechanistically independent of NHR-68. Therefore, we do not directly link the *aex-5* phenotype to the NHR-68 pathway, and manipulations such as *fat-2* RNAi would not establish a mechanistic connection between *aex-5* and NHR-68-dependent avoidance. We have clarified this interpretation in the revised manuscript. Lines 277-282

Specific comments:

L51: "the entire spectrum of fatty acid molecules de novo" not clear to me what is meant by this - all fatty acids?
Response: To improve clarity, we replaced “the entire spectrum of fatty acid molecules” with “a broad range of fatty acids,” which more accurately describes the fatty-acid biosynthetic capacity of *C. elegans.* Lines 58-60

L52: "In addition to their roles in energy storage [18]" This seems like an odd paper to cite here. 
Response: We removed “energy storage” and revised the sentence to “In addition to their roles in nervous system function, both monounsaturated fatty acids (MUFAs) and polyunsaturated fatty acids (PUFAs) can exhibit antibacterial functions.” Lines 60-62

L162 - "Vitamin B12 did not alter *clec-60* expression in *nhr-68(gk708)* animals (Fig S3A), indicating that the immune regulatory function of NHR-68 is independent of vitamin B12 metabolism."  - these could use a positive control, can they show that the B12 had the intended effect, e.g. decreased Pacdh-1::gfp?
Response: We thank the reviewer for this suggestion. The purpose of this experiment was to determine whether the immune regulatory function of NHR-68 depends on vitamin B12, rather than to characterize the physiological response to vitamin B12 itself. Because vitamin B12-mediated regulation of *acdh-1* has been extensively established in previous studies, we did not perform an additional *Pacdh-1::gfp* positive control assay in this study. To avoid overinterpretation, we have revised the text to clarify that our data indicate that vitamin B12 supplementation does not alter NHR-68-dependent regulation of *clec-60* expression under the conditions tested, rather than concluding a broader independence from vitamin B12 metabolism. Lines 185-186

L175 - "consistent with the previously described metabolic persistence detection module [29]." It's not clear to me how the results presented are consistent with this existing model, is it simply because the two genes are acting in parallel? 
Response: We thank the reviewer for pointing this out. To better align our interpretation with the experimental data, we revised the text to more conservatively state that NHR-10 and NHR-68 both contribute to the regulation of molecular and behavioral immune responses and removed the statement linking our findings to the previously described metabolic persistence detection module. Lines 192-195

L230 - "suppressed the increased survival in *nhr-68(gk708)* animals infected with *P. aeruginosa* PA14 (Figs 3D-3F)." This result could use some kind of statistical comparison - seems like an effect in the daf-16 background at least. 
Response: In the revised manuscript, we have replaced “suppressed” with “reduced the magnitude of the phenotype” and revised the conclusion to more conservatively state that PMK-1 and DAF-16 contribute to the increased survival phenotype of *nhr-68(gk708)* animals during *P. aeruginosa* PA14 infection. We have also clarified in the figure legend that statistical comparisons were performed within each RNAi condition. Lines 225-229

L239 - ". Given that alterations in the defecation motor program (DMP), such as those induced by *aex-5* RNAi [26], can lead to intestinal distension and enhanced pathogen avoidance, we utilized this model to identify metabolic changes potentially associated with avoidance behavior." This is confusing, there are many genetic manipulations that alter pathogen avoidance, was this background used simply to identify LA but is subsequently unrelated to the nhr-68 context? 
Response: We thank the reviewer for this comment. We have revised the text to clarify that *aex-5* RNAi was used as a functional discovery model for metabolite identification, and updated the manuscript accordingly. Lines 277-282

GC-MS - can the authors please clear up how they ended up with the short, finalized list of metabolites from the GCMS analysis? Also, why is linoleic acid listed twice in the dataset S5? 
Multiple places - it's implied and shown in the model that LA doesn't affect the immune response, but this doesn't seem to have been specifically tested.

Response: We clarify that the final metabolite list in the GC–MS dataset was previously generated based on a >2-fold change threshold for differential abundance. In the revised manuscript, we have now included the full metabolite dataset in updated Table S5 to improve transparency.

Regarding Table S5, linoleic acid is not duplicated. The previously listed entries correspond to distinct metabolites: line 4 represents linoleic acid (18:2 n-6), whereas line 5 corresponds to γ-linolenic acid (18:3 n-6), which differ by one additional double bond and belong to sequential steps in the ω-6 fatty acid biosynthetic pathway.

We performed additional PA14 survival assays in neuron-ablated backgrounds and under metabolic perturbations that modulate LA levels, including LA supplementation and *fat-3* RNAi. Although these manipulations alter avoidance behavior, none affected the enhanced survival phenotype of *nhr-68(gk708)* animals (new Figs S8A-S8B), supporting a model in which LA primarily regulates behavioral avoidance rather than survival outcomes. Lines 322-325 and 342-346

The methods and the text are a little unclear in places, this could be cleaned up a bit. 
Statistical comparisons - If the authors are reliant on significance testing for their interpretations, it seems odd to me why comparisons have been performed in some cases e.g. 4C, LA treatment across genotypes, but Fig 5 there are no comparisons across genotypes - many other cases of this.

Response: We agree that additional clarification is helpful. Statistical comparisons were performed only for pre-specified, biologically relevant contrasts that were directly related to the questions being addressed in each experiment. Therefore, not all possible cross-genotype comparisons were performed (including in Fig 5), and significance testing was not intended for all possible group combinations.

Citations - There are several places where the work of Zhang, Kim etc… on pathogen avoidance should be cited. Also the work of Mukhopadhay, Watts etc.. about PUFA supplementation and metabolism should be discussed. O'Halloran showed that *fat-3* acts cell autonomously in AWC for adaptation, how does this fit into the model?

Response: We have added the relevant references on pathogen avoidance and PUFA metabolism and expanded the Discussion to incorporate the previous finding that FAT-3 functions cell autonomously in AWC neurons and how it relates to our proposed model. Lines 53-57, 500-505, and 561-564
