# Supplementary material for: Intestinal lipid metabolism controls molecular and behavioral immunity through NHR-68 and gut–brain signaling in Caenorhabditis elegans
Source: PLoS Biol. 2026 Sep 29;24(9):e3004026. doi: 10.1371/journal.pbio.3004026 (PMC13649124; doi:10.1371/journal.pbio.3004026)
Supplement: Supplementary file 4 [file pbio.3004026.s012.docx]

Response to Reviewers

**Reviewer #3**

The authors had addressed some of my comments with additional experiments (major comments 1, 5, 7 and 9) or explanation (major comments 6, 8; minor comments 1, 4 and 6). They did not address major comments 2, 3 and 4, considering them to be out of the study scope. They also missed minor comments 3 and 5. Overall, with these partial revisions, the manuscript was improved so that the new findings and limitation in interpretation were both presented clearly. Below are some comments that the authors could further consider.

Response: We thank the reviewer for the careful review of our manuscript. We have addressed the remaining comments and revised the manuscript accordingly.

1. Figure 4G: Is the level of LA significantly increased under *Pseudomonas aeruginosa* infection? Data here is shown in colors but the authors should use a more quantitative format to present these results, and perform statistical analysis to explore whether LA levels with *Pseudomonas* infection are significantly higher than those in the control animals.

Response: We thank the reviewer for this suggestion. To better present these results, we retained Fig 4G and added a new Fig 4H showing mean ± SD of LA abundance. One-way ANOVA confirmed that exposure to *P. aeruginosa* significantly increased LA abundance. We also provided raw MS data in Table S5.

2. For my major comment 9, the authors only quantified reversals for the *nhr-10* and *nhr-68* mutants, as well as those for the double mutant. The locomotion speed should also be evaluated.

Response: We added locomotion speed analysis of WT, *nhr-10(tm4695)*, *nhr-68(gk708),* and *nhr-10(tm4695)*;*nhr-68(gk708)* animals. No significant differences were detected among groups, indicating that the reduced avoidance phenotype was not due to impaired locomotion. Lines 145-149

3. I still think it better to incorporate the data of Figure 3D Figure into Figure 3E and 3F.

Response: We have incorporated the data from Fig 3D into Figs 3E and 3F (new Figs 3D and 3E).

4. I do not agree that "immune response" in the title includes behavioral avoidance. This is certainly authors' personal take and not the standard practice of the field.

Response: We thank the reviewer for this suggestion. We agree and have revised the title to: “Intestinal lipid metabolism controls molecular and behavioral immunity through NHR-68 and gut–brain signaling in *C. elegans*”.

**Reviewer #4**

The authors have satisfactorily addressed all of my specific concerns in this revision.

Response: We thank the reviewer for the careful review of our manuscript and for the positive assessment of our revisions.

Minor note - the revised manuscript results refers to an intestine specific function of *nhr-68* prior to presenting the intestinal rescue in figure 6.  e.g. line 397 "AWC functions…downstream of intestinal metabolic regulation."  I would suggest rephrasing for clarity before publication.

Response: We have revised the text to avoid implying an intestine-specific function of NHR-68 before presenting the intestinal rescue data. The revised sentence is: “Together, these data suggest that AWC neurons are required for both NHR-68-regulated and PUFA-induced avoidance of *P. aeruginosa*, indicating that AWC functions as a consistent component of the behavioral circuit involved in this response.” Line 398
