# Supplementary material for: Intestinal lipid metabolism controls molecular and behavioral immunity through NHR-68 and gut–brain signaling in Caenorhabditis elegans
Source: PLoS Biol. 2026 Sep 29;24(9):e3004026. doi: 10.1371/journal.pbio.3004026 (PMC13649124; doi:10.1371/journal.pbio.3004026)
Supplement: Peer review history

# Author comment: Author response to Decision Letter 0

**DOI:** 10.1371/journal.pbio.3004026.r001

22 Jun 2026

Attachment Submitted filename: Point-by-point response .docx

# Editor report: Decision Letter 0

**DOI:** 10.1371/journal.pbio.3004026.r002

**Authors:** Lucas Smith

30 Jun 2026

Dear Dr Sang,

Thank you for submitting your revised manuscript entitled "Intestinal lipid metabolism controls immune response through NHR-68 and gut–brain signaling in C. elegans" for consideration as a Research Article by PLOS Biology. I have now had a chance to discuss your revision with the Academic Editor, and I am writing to let you know that we would like to send your submission back to the original reviewers for another look.

However, before we can send your manuscript to reviewers, we need you to complete your submission by providing the metadata that is required for full assessment. To this end, please login to Editorial Manager where you will find the paper in the 'Submissions Needing Revisions' folder on your homepage. Please click 'Revise Submission' from the Action Links and complete all additional questions in the submission questionnaire.

Once your full submission is complete, your paper will undergo a series of checks in preparation for peer review. After your manuscript has passed the checks it will be sent out for review. To provide the metadata for your submission, please Login to Editorial Manager (https://www.editorialmanager.com/pbiology) within two working days, i.e. by Jul 02 2026 11:59PM.

Feel free to email us at plosbiology@plos.org if you have any queries relating to your submission.

Kind regards,

Luke

Lucas Smith, Ph.D.

Senior Editor

PLOS Biology

lsmith@plos.org

# Author comment: Author response to Decision Letter 1

**DOI:** 10.1371/journal.pbio.3004026.r003

1 Jul 2026

Attachment Submitted filename: Point-by-point_response__auresp_1.docx

# Peer review: Decision Letter 1

**DOI:** 10.1371/journal.pbio.3004026.r004

**Authors:** Lucas Smith

4 Aug 2026

Dear Dr Sang,

Thank you for your patience while we considered your revised manuscript "Intestinal lipid metabolism controls immune response through NHR-68 and gut–brain signaling in C. elegans" for consideration as a Research Article at PLOS Biology. Your revised study has now been evaluated by the PLOS Biology editors, the Academic Editor and by two of the original reviewers.

In light of the reviews, which you will find at the end of this email, we are pleased to offer you the opportunity to address the remaining points from the reviewers in a revision that we anticipate should not take you very long. We will then assess your revised manuscript and your response to the reviewers' comments with our Academic Editor aiming to avoid further rounds of peer-review, although we might need to consult with the reviewers, depending on the nature of the revisions.

In addition to these revisions, please be sure to also respond to our editorial requests, which are appended below the reviews.

You may also need to complete some formatting changes, which you will receive in a follow up email. A member of our team will be in touch with a set of requests shortly. If you do not receive a separate email within a few days, please assume that checks have been completed, and no additional changes are required.

We expect to receive your revised manuscript within 1 month. Please email us (plosbiology@plos.org) if you have any questions or concerns, or would like to request an extension.

At this stage, your manuscript remains formally under active consideration at our journal; please notify us by email if you do not intend to submit a revision so that we withdraw the manuscript.

**IMPORTANT - SUBMITTING YOUR REVISION**

Your revisions should address the specific points made by each reviewer. Please submit the following files along with your revised manuscript:

1. A 'Response to Reviewers' file - this should detail your responses to the editorial requests, present a point-by-point response to all of the reviewers' comments, and indicate the changes made to the manuscript.

*NOTE: In your point-by-point response to the reviewers, please provide the full context of each review. Do not selectively quote paragraphs or sentences to reply to. The entire set of reviewer comments should be present in full and each specific point should be responded to individually.

You should also cite any additional relevant literature that has been published since the original submission and mention any additional citations in your response.

2. In addition to a clean copy of the manuscript, please also upload a 'track-changes' version of your manuscript that specifies the edits made. This should be uploaded as a "Revised Article with Changes Highlighted " file type.

*Resubmission Checklist*

When you are ready to resubmit your revised manuscript, please refer to this resubmission checklist: https://plos.io/Biology_Checklist

To submit a revised version of your manuscript, please go to https://www.editorialmanager.com/pbiology/ and log in as an Author. Click the link labelled 'Submissions Needing Revision' where you will find your submission record.

Please make sure to read the following important policies and guidelines while preparing your revision:

*Published Peer Review*

Please note while forming your response, if your article is accepted, you may have the opportunity to make the peer review history publicly available. The record will include editor decision letters (with reviews) and your responses to reviewer comments. If eligible, we will contact you to opt in or out. Please see here for more details:

https://blogs.plos.org/plos/2019/05/plos-journals-now-open-for-published-peer-review/

*PLOS Data Policy*

Please note that as a condition of publication PLOS' data policy (http://journals.plos.org/plosbiology/s/data-availability) requires that you make available all data used to draw the conclusions arrived at in your manuscript. If you have not already done so, you must include any data used in your manuscript either in appropriate repositories, within the body of the manuscript, or as supporting information (N.B. this includes any numerical values that were used to generate graphs, histograms etc.). For an example see here: http://www.plosbiology.org/article/info%3Adoi%2F10.1371%2Fjournal.pbio.1001908#s5

*Blot and Gel Data Policy*

We require the original, uncropped and minimally adjusted images supporting all blot and gel results reported in an article's figures or Supporting Information files. We will require these files before a manuscript can be accepted so please prepare them now, if you have not already uploaded them. Please carefully read our guidelines for how to prepare and upload this data: https://journals.plos.org/plosbiology/s/figures#loc-blot-and-gel-reporting-requirements

*Protocols deposition*

To enhance the reproducibility of your results, we recommend that if applicable you deposit your laboratory protocols in protocols.io, where a protocol can be assigned its own identifier (DOI) such that it can be cited independently in the future. Additionally, PLOS ONE offers an option for publishing peer-reviewed Lab Protocol articles, which describe protocols hosted on protocols.io. Read more information on sharing protocols at https://plos.org/protocols?utm_medium=editorial-email&utm_source=authorletters&utm_campaign=protocols

Thank you again for your submission to our journal. We hope that our editorial process has been constructive thus far, and we welcome your feedback at any time. Please don't hesitate to contact us if you have any questions or comments.

Sincerely,

Luke

Lucas Smith, Ph.D.

Senior Editor

PLOS Biology

lsmith@plos.org

----------------------------------------------------------------

REVIEWS:

Reviewer #3: The authors had addressed some of my comments with additional experiments (major comments 1, 5, 7 and 9) or explanation (major comments 6, 8; minor comments 1, 4 and 6). They did not address major comments 2, 3 and 4, considering them to be out of the study scope. They also missed minor comments 3 and 5. Overall, with these partial revisions, the manuscript was improved so that the new findings and limitation in interpretation were both presented clearly. Below are some comments that the authors could further consider.

1. Figure 4G: Is the level of LA significantly increased under Pseudomonas aeruginosa infection? Data here is shown in colors but the authors should use a more quantitative format to present these results, and perform statistical analysis to explore whether LA levels with Pseudomonas infection are significantly higher than those in the control animals.

2 For my major comment 9, the authors only quantified reversals for the nhr-10 and nhr-68 mutants, as well as those for the double mutant. The locomotion speed should also be evaluated.

3. I still think it better to incorporate the data of Figure 3D Figure into Figure 3E and 3F.

4. I do not agree that "immune response" in the title includes behavioral avoidance. This is certainly authors' personal take and not the standard practice of the field.

Reviewer #4: The authors have satisfactorily addressed all of my specific concerns in this revision. Minor note - the revised manuscript results refers to an intestine specific function of nhr-68 prior to presenting the intestinal rescue in figure 6. e.g. line 397 "AWC functions…downstream of intestinal metabolic regulation." I would suggest rephrasing for clarity before publication.

EDITORIAL REQUESTS

1) FINANCIAL DISCLOSURES: Please update the financial disclosures statement in our online editorial manager system to indicate whether the sponsors or funders played any role in the study design, data collection and analysis, decision to publish, or preparation of the manuscript.

2) DATA: You may be aware of the PLOS Data Policy, which requires that all data be made available without restriction: http://journals.plos.org/plosbiology/s/data-availability. For more information, please also see this editorial: http://dx.doi.org/10.1371/journal.pbio.1001797

>>Please upload all raw RNA-seq and mass spec (GC-MS and LC-HRMS) data to publicly available repositories.

Note that for other figures we do not require all raw data. Rather, we ask that all individual quantitative observations that underlie the data summarized in the figures and results of your paper be made available in one of the following forms:

a. Supplementary files (e.g., excel). Please ensure that all data files are uploaded as 'Supporting Information' and are invariably referred to (in the manuscript, figure legends, and the Description field when uploading your files) using the following format verbatim: S1 Data, S2 Data, etc. Multiple panels of a single or even several figures can be included as multiple sheets in one excel file that is saved using exactly the following convention: S1_Data.xlsx (using an underscore).

b. Deposition in a publicly available repository. Please also provide the accession code or a reviewer link so that we may view your data before publication.

>>Regardless of the method selected, please ensure that you provide the individual numerical values that underlie the summary data displayed in your figures (including supplemental)

NOTE: the numerical data provided should include all replicates AND the way in which the plotted mean and errors were derived (it should not present only the mean/average values).

>>Please also ensure that figure legends in your manuscript include information on where the underlying data can be found, and ensure your supplemental data file/s has a legend.

>>Please ensure that your Data Statement in the submission system accurately describes where your data can be found.

3) DATA PRESENTATION: Please ensure that you are using best practice for statistical reporting and data presentation. These are our guidelines https://journals.plos.org/plosbiology/s/best-practices-in-research-reporting#loc-statistical-reporting and a useful resource on data presentation https://journals.plos.org/plosbiology/article?id=10.1371/journal.pbio.1002128

>>If you are reporting experiments where n ≤ 5, please plot each individual data point.\

4) CODE: Per journal policy, if you have generated any custom code during the course of this investigation, please make it available without restrictions. Please ensure that the code is sufficiently well documented and reusable, and that your Data Statement in the Editorial Manager submission system accurately describes where your code can be found. More information on our Code Policy, what and how to share can be found here: https://journals.plos.org/plosbiology/s/code-availability

Please note that we cannot accept sole deposition of code in GitHub, as this could be changed after publication. However, you can archive this version of your publicly available GitHub code to Zenodo. Once you do this, it will generate a DOI number, which you will need to provide in the Data Accessibility Statement (you are welcome to also provide the GitHub access information). See the process for doing this here: https://docs.github.com/en/repositories/archiving-a-github-repository/referencing-and-citing-content

# Author comment: Author response to Decision Letter 2

**DOI:** 10.1371/journal.pbio.3004026.r005

12 Aug 2026

Attachment Submitted filename: Response to Reviewers.docx

# Editor report: Decision Letter 2

**DOI:** 10.1371/journal.pbio.3004026.r006

**Authors:** Lucas Smith

28 Aug 2026

Dear Dr Sang,

Thank you for your patience while we considered your revised manuscript "Intestinal lipid metabolism controls molecular and behavioral immunity through NHR-68 and gut–brain signaling in C. elegans" for publication as a Research Article at PLOS Biology. This revised version of your manuscript has been evaluated by the PLOS Biology editors and the Academic Editor, who is satisfied by the changes made in response to the last round of review.

Based on our Academic Editor's assessment of your revision we are likely to accept this manuscript for publication. However before we can accept your study we need you to address a number of last editorial requests, which I think may have been missed in my previous decision letter. These are detailed below.

**IMPORTANT - Please address the following editorial requests.

1) DATA: In my last email, I asked that you please upload all raw RNA-seq and mass spec (GC-MS and LC-HRMS) data to publicly available repositories. Looking through the revision, I could not find your response to this request, or any accession numbers. Did I miss them somewhere? If not, please upload the raw data (ex FASTQ files, vendor raw files and open-format converted files) together with the metadata needed to reproduce the processing for all samples profiled by these techniques, as that is standard practice in the field, and is needed to meet our data reporting policy (http://journals.plos.org/plosbiology/s/data-availability)

2) DATA: In addition to the data requested above, for the rest of the figures presented in your study we ask that you provide the individual quantitative observations that underlie the presented data. This can be made available in the following forms:

a. Supplementary files (e.g., excel). Please ensure that all data files are uploaded as 'Supporting Information' and are invariably referred to (in the manuscript, figure legends, and the Description field when uploading your files) using the following format verbatim: S1 Data, S2 Data, etc. Multiple panels of a single or even several figures can be included as multiple sheets in one excel file that is saved using exactly the following convention: S1_Data.xlsx (using an underscore).

b. Deposition in a publicly available repository. Please also provide the accession code or a reviewer link so that we may view your data before publication.

>>Regardless of the method selected, please ensure that you provide the individual numerical values that underlie the summary data displayed in the following figures:

Fig 1B-D; Fig 2B-D; Fig 3C-E; Fig C-J; Fig 5A-H; Fig 6A-H;

Fig S1; Fig S2A-D; Fig S3A-B; Fig S6; Fig S7; Fig S8l Fig S9; Fig S10

NOTE: the numerical data provided should include all replicates AND the way in which the plotted mean and errors were derived (it should not present only the mean/average values).

>>Please also ensure that figure legends in your manuscript include information on where the underlying data can be found, and ensure your supplemental data file/s has a legend.

>>Please ensure that your Data Statement in the submission system accurately describes where your data can be found.

^re: this request, I do see that your study does have some supplemental tables that may include relevant data, but I had trouble finding most of the underlying data (so I apologize if I missed some of the data already).

3) DATA PRESENTATION: In my previous email I asked that 'if you are reporting experiments where n ≤ 5, please plot each individual data point' - however I noticed a few figures that do not meet this requirement. So please do take a moment to double check that your figures meet the best practice for statistical reporting and data presentation. These are our guidelines https://journals.plos.org/plosbiology/s/best-practices-in-research-reporting#loc-statistical-reporting and a useful resource on data presentation https://journals.plos.org/plosbiology/article?id=10.1371/journal.pbio.1002128

When checking this point, I also saw a few figure legends where the 'n's seemed to be missing, and so I would encourage you to do a final check the figure legends (including supplemental) to make sure they contain all the relevant details about sample size and stats, etc.

4) CODE: I also did not see your response to my request for code - so I am re-iterating it here, in case you generated any code in this study:

Per journal policy, if you have generated any custom code during the course of this investigation, please make it available without restrictions. Please ensure that the code is sufficiently well documented and reusable, and that your Data Statement in the Editorial Manager submission system accurately describes where your code can be found. More information on our Code Policy, what and how to share can be found here: https://journals.plos.org/plosbiology/s/code-availability

Please note that we cannot accept sole deposition of code in GitHub, as this could be changed after publication. However, you can archive this version of your publicly available GitHub code to Zenodo. Once you do this, it will generate a DOI number, which you will need to provide in the Data Accessibility Statement (you are welcome to also provide the GitHub access information). See the process for doing this here: https://docs.github.com/en/repositories/archiving-a-github-repository/referencing-and-citing-content

As you address these items, please take this last chance to review your reference list to ensure that it is complete and correct. If you have cited papers that have been retracted, please include the rationale for doing so in the manuscript text, or remove these references and replace them with relevant current references. Any changes to the reference list should be mentioned in the cover letter that accompanies your revised manuscript.

In addition to these revisions, you may need to complete some formatting changes, which you will receive in a follow up email. A member of our team will be in touch with a set of requests shortly. If you do not receive a separate email within a few days, please assume that checks have been completed, and no additional changes are required.

We expect to receive your revised manuscript within two weeks.

To submit your revision, please go to https://www.editorialmanager.com/pbiology/ and log in as an Author. Click the link labelled 'Submissions Needing Revision' to find your submission record. Your revised submission must include the following:

- a cover letter that should detail your responses to any editorial requests, if applicable, and whether changes have been made to the reference list

- a Response to Reviewers file that provides a detailed response to the reviewers' comments (if applicable, if not applicable please do not delete your existing 'Response to Reviewers' file.)

- a track-changes file indicating any changes that you have made to the manuscript.

NOTE: If Supporting Information files are included with your article, note that these are not copyedited and will be published as they are submitted. Please ensure that these files are legible and of high quality (at least 300 dpi) in an easily accessible file format. For this reason, please be aware that any references listed in an SI file will not be indexed. For more information, see our Supporting Information guidelines:

https://journals.plos.org/plosbiology/s/supporting-information

*Published Peer Review History*

Please note that you may have the opportunity to make the peer review history publicly available. The record will include editor decision letters (with reviews) and your responses to reviewer comments. If eligible, we will contact you to opt in or out. Please see here for more details:

https://plos.org/published-peer-review-history/

*Press*

Should you, your institution's press office or the journal office choose to press release your paper, please ensure you have opted out of Early Article Posting on the submission form. We ask that you notify us as soon as possible if you or your institution is planning to press release the article.

*Protocols deposition*

To enhance the reproducibility of your results, we recommend that if applicable you deposit your laboratory protocols in protocols.io, where a protocol can be assigned its own identifier (DOI) such that it can be cited independently in the future. Additionally, PLOS ONE offers an option for publishing peer-reviewed Lab Protocol articles, which describe protocols hosted on protocols.io. Read more information on sharing protocols at https://plos.org/protocols?utm_medium=editorial-email&utm_source=authorletters&utm_campaign=protocols

Please do not hesitate to contact me should you have any questions.

Sincerely,

Luke

Lucas Smith, Ph.D.

Senior Editor

lsmith@plos.org

PLOS Biology

# Editor report: Decision Letter 3

**DOI:** 10.1371/journal.pbio.3004026.r007

**Authors:** Lucas Smith

14 Sep 2026

Dear Dr Sang,

Thank you for the submission of your revised Research Article "Intestinal lipid metabolism controls molecular and behavioral immunity through NHR-68 and gut–brain signaling in C. elegans" for publication in PLOS Biology and thank you for addressing our last editorial requests in this revision. On behalf of my colleagues and the Academic Editor, Mark J Alkema , I am pleased to say that we can in principle accept your manuscript for publication, provided you address any remaining formatting and reporting issues. These will be detailed in an email you should receive within 2-3 business days from our colleagues in the journal operations team; no action is required from you until then. Please note that we will not be able to formally accept your manuscript and schedule it for publication until you have completed any requested changes.

**IMPORTANT- In addition to addressing any production requests, to come, please be sure to address the following editorial requests.

1) As discussed over email, I have updated your submission to include the updated S1_data and supporting figures files that you provided me. Please take a moment to look through your submission to make sure the changes all look OK.

2) I have also taken the liberty of updating the data and code availability statement, in our online editorial manager system, to include the version that you provided in your manuscript, as the online version is the one that will be published with your paper. Note, I slightly tweaked it, so please make sure the statement also looks good to you. It reads:

The raw RNA-seq data have been deposited in the NCBI Gene Expression Omnibus (GEO) database under accession number GSE344153.

The raw GC-MS metabolomics data have been deposited in MassIVE under accession number: MSV000102834.

The raw LC-HRMS metabolomics data have been deposited in the Metabolomics Workbench under study ID:ST005144.

No custom code was generated for this study.

All other underlying data for this study are available within the paper and its Supporting Information files

3) Please update the figure legend for supplemental figure 1 to include the statement "The underlying data for this figure can be found in S1 data."

4) As discussed over email, please do update all figures, excluding lifespan panels, (but including the supplemental figures) with n ≤ 5 to show the individual data points. I will ask the production team to loop me in once this change is made.

Please take a minute to log into Editorial Manager at http://www.editorialmanager.com/pbiology/, click the "Update My Information" link at the top of the page, and update your user information to ensure an efficient production process.

PRESS

We frequently collaborate with press offices. If your institution or institutions have a press office, please notify them about your upcoming paper at this point, to enable them to help maximise its impact. If the press office is planning to promote your findings, we would be grateful if they could coordinate with biologypress@plos.org. If you have previously opted in to the early version process, we ask that you notify us immediately of any press plans so that we may opt out on your behalf.

We also ask that you take this opportunity to read our Embargo Policy regarding the discussion, promotion and media coverage of work that is yet to be published by PLOS. As your manuscript is not yet published, it is bound by the conditions of our Embargo Policy. Please be aware that this policy is in place both to ensure that any press coverage of your article is fully substantiated and to provide a direct link between such coverage and the published work. For full details of our Embargo Policy, please visit http://www.plos.org/about/media-inquiries/embargo-policy/.

Thank you again for choosing PLOS Biology for publication and supporting Open Access publishing. We look forward to publishing your study.

Sincerely,

Luke

Lucas Smith, Ph.D.

Senior Editor

PLOS Biology

lsmith@plos.org
